# An Unusual Presentation of Myocardial Ischemia and Coexisting Asthma Exacerbation With a Pseudo-Wellens Sign: A Case Report

**DOI:** 10.7759/cureus.87110

**Published:** 2025-07-01

**Authors:** Can Baba Arin

**Affiliations:** 1 Cardiology, Health Sciences University, Dr. Siyami Ersek Thoracic and Cardiovascular Surgery Training and Research Hospital, Istanbul, TUR

**Keywords:** asthma exacerbation, cardiac biomarkers, ecg, myocardial ischemia, pseudo-wellens sign

## Abstract

Cardiovascular complications, including myocardial ischemia, can occur during acute asthma exacerbations due to overlapping inflammatory and physiological mechanisms. ECG abnormalities and elevated cardiac biomarkers may mimic acute coronary syndrome, complicating diagnosis and management. We present a case of a 60-year-old woman with a history of long-standing asthma and hypertension who presented with chest pain and shortness of breath during an asthma exacerbation. Clinical evaluation revealed diffuse wheezing, deep T-wave inversions on ECG, and elevated cardiac biomarkers, initially raising concern for Wellens syndrome. Transthoracic echocardiography showed mild left ventricular hypertrophy and regional wall motion abnormalities, but coronary angiography demonstrated no obstructive coronary artery disease. The patient’s symptoms and oxygen saturation improved with bronchodilator and corticosteroid therapy, supporting a diagnosis of pseudo-Wellens syndrome in the setting of status asthmaticus. This case highlights the importance of recognizing pseudo-Wellens syndrome as a potential mimic of true myocardial ischemia to avoid unnecessary invasive procedures and guide appropriate treatment.

## Introduction

The association between cardiovascular complications, such as myocardial ischemia, and asthma exacerbations is well established. Multiple studies have explored this relationship, highlighting how the cardiac and pulmonary systems influence one another during acute illness. Patients presenting with ECG abnormalities suggestive of myocardial ischemia often have elevated cardiac biomarkers [1]. Ischemic ECG patterns and elevated troponin levels have been reported in both pediatric and adult patients undergoing asthma treatment and may mimic acute coronary syndrome [2].

Although Wellens syndrome is characterized by a distinctive ECG pattern, other conditions can produce similar findings. Pseudo-Wellens syndrome replicates these ECG changes but occurs in the absence of significant coronary artery disease and may be associated with acute myocardial ischemia or coronary vasospasm [3]. Myocardial dysfunction during the management of acute asthma has also been reported, potentially triggered by beta-agonists, particularly in individuals with underlying cardiovascular disease (CVD) [4]. This transient myocardial impairment often resolves with effective asthma treatment, suggesting a direct relationship between status asthmaticus and cardiac dysfunction. One case described a patient with long-standing asthma who developed coronary vasospasm during a severe exacerbation, ultimately progressing to cardiogenic shock and recurrent ventricular fibrillation [5]. This report describes a case of a woman with long-standing asthma who presented with chest pain and ECG changes suggestive of Wellens syndrome, ultimately diagnosed as pseudo-Wellens syndrome in the absence of obstructive coronary artery disease.

## Case presentation

A 60-year-old woman presented to the emergency department with shortness of breath and chest pain. Her medical history included asthma and hypertension. The patient’s cardiovascular risk factors included a BMI of 31 kg/m², indicating obesity, and a low-density lipoprotein (LDL) cholesterol level of 132 mg/dL. She denied smoking or alcohol use. There was no known family history of premature coronary artery disease. Her asthma had been longstanding, with frequent exacerbations requiring systemic steroids in the past year. Maintenance therapy included a combination of a long-acting beta-agonist and an inhaled corticosteroid. No recent pulmonary function testing or baseline spirometry was available. Her medications included amlodipine 10 mg and a combination inhaler containing formoterol fumarate 12 mcg and budesonide 400 mcg.

**Figure 1:**
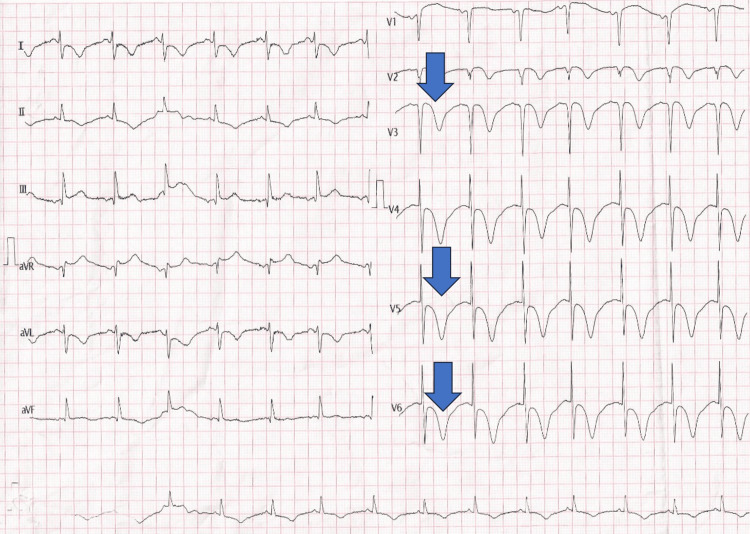
Twelve-lead electrocardiogram showing normal sinus rhythm with deep, symmetric T-wave inversions in the precordial leads V1-V4, consistent with a pseudo-Wellens pattern. Findings are suggestive of a pseudo-Wellens sign in a 60-year-old female with an asthma exacerbation.

On examination, the patient was alert and oriented. Vital signs were as follows: blood pressure, 140/85 mmHg; pulse, 80 beats per minute; respiratory rate, 35 breaths per minute; and oxygen saturation, 90% on room air. Diffuse wheezing was noted on auscultation. ECG showed normal sinus rhythm with deep T-wave inversions in the precordial leads (Figure 1).

Transthoracic echocardiography revealed mild left ventricular hypertrophy and hypokinesia in the left anterior descending (LAD) coronary artery territory. A follow-up transthoracic echocardiogram performed 48 hours later showed resolution of the previously noted regional wall-motion abnormalities in the LAD territory, consistent with transient myocardial stunning rather than infarction. No pericardial effusion or structural abnormalities were observed. Laboratory studies showed elevated creatine kinase-MB (CK-MB) fraction (63 U/L; reference range: 1-24 U/L) and troponin (2,222 ng/mL; reference range: 0.02-0.06 ng/mL). Serial cardiac biomarkers showed a rising and then falling pattern, consistent with transient myocardial injury. Troponin I was 1,796 ng/mL at admission, peaked at 2,222 ng/mL after three hours, declined to 980 ng/mL following coronary angiography, and further dropped to 0.95 ng/mL at 48 hours. These trends suggest a non-infarct etiology. Her CK-MB levels were elevated initially but also normalized over time.

**Figure 2:**
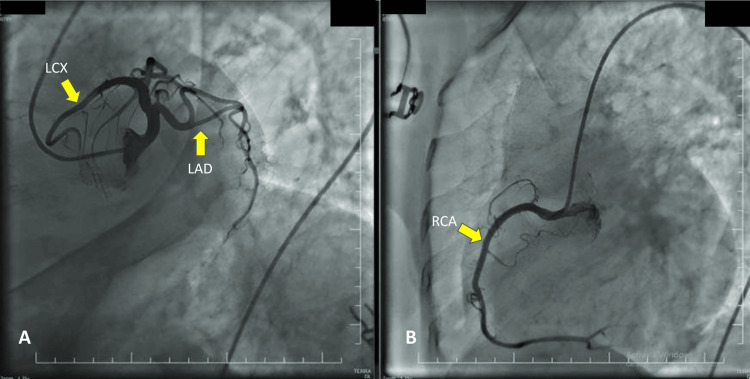
Coronary angiography demonstrating no evidence of obstructive disease in the left anterior descending, left circumflex, or right coronary arteries. LAD: Left anterior descending artery; LCx: Left circumflex artery; RCA: Right coronary artery. No obstructive lesions were observed in any of the three main coronary vessels.

The patient was treated with an ipratropium/albuterol inhaler and received 40 mg of IV methylprednisolone. Her symptoms improved, and her oxygen saturation increased to 96%. Despite the continued elevation of cardiac biomarkers after eight hours, coronary angiography revealed no evidence of obstructive coronary artery disease (Figure 2). She was discharged on the fifth hospital day with a treatment plan that included amlodipine/valsartan 10/160 mg, isosorbide mononitrate 20 mg, and continuation of her asthma therapy.

## Discussion

Myocardial ischemia and asthma are linked through shared inflammatory pathways and physiological stress responses. Asthma, characterized by chronic airway inflammation, has been identified as an independent risk factor for CVD [3,5].

Severe asthma exacerbations can cause hypoxia and bronchospasm, both of which may compromise myocardial perfusion and contribute to ischemia. Bronchospasm-induced hypoxia has been associated with coronary vasospasm and arrhythmias during asthma attacks [4,6].

Conversely, preexisting CVD may worsen asthma control, creating a bidirectional exacerbation of both conditions [6]. In this case, the patient’s ECG findings resembled those seen in Wellens syndrome; however, coronary angiography revealed no significant stenosis. This presentation is consistent with pseudo-Wellens syndrome, a transient and reversible condition that mimics acute coronary syndrome on ECG and biomarkers but does not reflect true atherosclerotic plaque rupture or occlusion. Pseudo-Wellens syndrome has also been observed in pulmonary and other non-atherosclerotic conditions [4,7,8]. Recognizing such mimics is vital.

The extremely elevated cardiac troponin levels observed in this case, peaking at over 2,200 ng/mL, presented a diagnostic challenge. In the absence of angiographic evidence of obstructive coronary artery disease and with a favorable clinical course, the findings were most consistent with transient myocardial injury rather than true infarction. Several pathophysiological mechanisms have been implicated in such presentations. These include β2-agonist-induced myocardial stress, which can increase heart rate and myocardial oxygen demand [1]; hypoxia-mediated coronary vasospasm resulting from severe bronchospasm and impaired ventilation [8]; and demand ischemia during status asthmaticus, especially in patients with underlying cardiovascular comorbidities [2]. Additionally, microvascular dysfunction or non-ischemic myocardial strain may contribute to myocardial injury without epicardial coronary obstruction. Cardiac MRI was not performed; however, the absence of late gadolinium enhancement, persistent wall motion abnormalities, or classic apical ballooning makes Takotsubo cardiomyopathy and myocarditis less likely.

## Conclusions

This case illustrates the diagnostic challenge of interpreting ischemic ECG changes in the context of an acute asthma exacerbation. Pseudo-Wellens syndrome can closely resemble true Wellens syndrome but occurs in the absence of obstructive coronary artery disease. Early recognition of this pattern is essential to prevent unnecessary invasive procedures and to ensure appropriate management. Clinicians should maintain a high index of suspicion for pseudo-Wellens syndrome, particularly in patients presenting with overlapping respiratory and cardiac symptoms.
